# Investigating DRD2 and HTR2A polymorphisms in treatment-resistant schizophrenia: a comparative analysis with other treatment-resistant mental disorders and the healthy state

**DOI:** 10.1007/s00406-025-01970-9

**Published:** 2025-02-12

**Authors:** Antonio Del Casale, Giovanna Gentile, Simone Lardani, Martina Nicole Modesti, Jan Francesco Arena, Clarissa Zocchi, Ottavia De Luca, Giovanna Parmigiani, Gloria Angeletti, Stefano Ferracuti, Robert Preissner, Maurizio Simmaco, Marina Borro, Maurizio Pompili

**Affiliations:** 1 Department of Dynamic and Clinical Psychology and Health Studies, Faculty of Medicine and Psychology, Sapienza University of Rome, Center for Precision Medicine, Sant’Andrea University Hospital, 00189 Rome, Italy; 2 Department of Neuroscience, Mental Health and Sensory Organs (NESMOS), Faculty of Medicine and Psychology, Sapienza University of Rome, Unit of Laboratory and Advanced Molecular Diagnostics, Center for Precision Medicine, ‘Sant’Andrea’ University Hospital, 00189 Rome, Italy; 3 Department of Neuroscience, Mental Health and Sensory Organs (NESMOS), Faculty of Medicine and Psychology, Sapienza University of Rome, Unit of Psychiatry, ‘Sant’Andrea’ University Hospital, 00189 Rome, Italy; 4 Department of Psychology, Faculty of Medicine and Psychology, Sapienza University of Rome, 00185 Rome, Italy; 5 Local Health Authority Rome 6, Mental Health Department, Mental Health Center - CSM Frascati, 00044 Frascati (RM), Italy; 6 Department of Human Neuroscience, Faculty of Medicine and Dentistry, Sapienza University of Rome, 00185 Rome, Italy; 7 Unit of Risk Management, Sant’Andrea University Hospital, 00189 Rome, Italy; 8 Structural Bioinformatics Group, Institute for Physiology, Charité—University Medicine Berlin, 10115 Berlin, Germany

**Keywords:** Schizophrenia treatment-resistant, Mental disorders, Haplotype, Dopamine D2 receptor, Serotonin 5-HT2A Receptor, Polymorphism genetic, Pharmacogenomics

## Abstract

This study investigates treatment-resistant schizophrenia (TRS) by analysing genetic markers in dopamine and serotonin receptors. Conducted on a cohort of 221 patients with treatment-resistant mental disorders, the research focused on DRD2 and HTR2A gene variants—specifically, rs1801028, rs6314, rs7997012, and rs6311. The findings suggest specific associations between certain genetic variants and TRS. Notably, the HTR2A rs6314 A|G genotype and rs7997012 G|G genotype were significantly more prevalent in TRS patients compared to healthy controls (HCs). Haplotype analyses revealed associations between specific haplotypes—such as A|G (rs6314-rs7997012)—and TRS, indicating their potential predictive value for TRS versus HCs. The study underscores the involvement of the serotonergic system in TRS. These findings offer valuable insights into the genetic factors contributing to TRS, paving the way for future research and the development of personalised prevention and treatment strategies in psychiatry.

## Introduction

Schizophrenia (SCZ) is a severe mental disorder with an incidence rate of about 1% in the general population [87]. The average age of onset is 20.5 years [76], with a male-to-female ratio of 1.4:1 [60]. The consensus is that the aetiology is multifactorial, with heritability contributing to 79% of the pathophysiology of this disorder [35].

Despite antipsychotics (APs) having proven effective in symptom control, 20–30% of affected individuals exhibit an unsatisfactory response to the available drugs, leading to a diagnosis of treatment-resistant schizophrenia (TRS) [12]. Criteria for TRS have been recognised since 1966 [40] and have been continuously updated. In 2017, the Treatment Response and Resistance in Psychosis (TRRIP) research group defined TRS as the failure of two different AP treatments, administered at an adequate dosage and for a sufficient duration [36]. Patients with TRS experience more organic comorbidities [23], more frequent suicidal ideation, poorer quality of life, and more severe cognitive impairment [39] than their treatment-responding counterparts, and they incur higher treatment costs [49].

Two hypotheses currently attempt to explain the neurobiological mechanisms underlying TRS. The first suggest that treatment resistance in SCZ is attributable to dysregulation in glutamatergic transmission, leading to hyperactivation of mesencephalic dopaminergic pathways directed to the striatum [29]. The second posits that hypersensitisation of D2 receptors to dopamine, resulting from the continuous receptor blockage operated by AP drugs, is the primary factor underlying TRS [9, 10].

Currently, the only drug approved as the gold-standard for TRS is clozapine [74]. Numerous studies have confirmed clozapine’s superiority over other APs in terms of symptom reduction and control of psychotic symptomatology [7, 83]. However, its use in clinical practice is limited by its tolerability profile, as it can cause tachycardia, metabolic syndrome, hypersalivation, and constipation, as well as agranulocytosis, pneumonia, myocarditis, and arrhythmias [14]. Despite its efficacy, 30% of patients show an insufficient response to clozapine [17], suggesting that the current clinical criteria for assessing response and resistance are inadequate for fully understanding the clinical entity of TRS [62]. Various factors can modulate clozapine's effects, including pharmacokinetic and pharmacodynamic interactions with other drugs, as well as genetic factors. At present, it is not possible to predict treatment efficacy in advance, making it difficult to select the most appropriate drug for individual patients. In this context, studying the pharmacogenomic profile of patients offers an opportunity to identify specific clinical populations that could benefit most from particular pharmacological treatments [52]. The present research focuses on pharmacogenomics as an advanced technique to enhance our understanding of treatment response in TRS and to improve treatment appropriateness.

In fact, pharmacogenomics is an interdisciplinary field that bridges pharmacology, genetics, and medicine, aiming to investigate how genetic heritability influences the response to pharmacological treatment. It represents a natural evolution of genetics, which focuses on studying genes associated with disease onset. This approach has paved the way for precision medicine, which seeks to improve patient safety, treatment efficacy, and overall health by using clinical markers to enhance risk stratification, tailor pharmacological interventions, and personalised treatment approaches [20]. Over 10% of drugs approved by the Food and Drug Administration (FDA) now include pharmacogenomic information in their datasheet, particularly regarding adverse reactions, dosage adjustments, or the need for pharmacological switches [47]. Genetic variants, such as single nucleotide polymorphisms (SNPs) could serve as pharmacogenomic markers to guide pharmaceutical prescriptions, potentially overcoming the current “trial and error” approach to drug selection [45].

In the case of TRS, current research is focusing on SNPs in genes potentially related to disease pathophysiology, such as those coding for dopamine or serotonin receptors [45]. The dopamine receptor D2, the primary target of most AP treatments [46], is encoded by the DRD2 gene located on human chromosome 11q23 [19]. Various studies have highlighted its possible role in the development of SCZ [44]. Among DRD2 SNPs, rs1800497 (also known as Taq1A), which influences the availability of dopaminergic receptors in the striatum [28], appears to play a role in TRS [84]. The A1 allele of Taq1A is associated with pathological addiction, antisocial behaviour, eating disorders and attention-deficit/hyperactivity disorder (ADHD), whereas the A2 allele is more commonly found in SCZ spectrum disorders and obsessive–compulsive disorder (OCD) [69]. Another significant SNP, rs1799732, has been associated with SCZ. This variant may lead to increased density of D2 receptors in the brain, resulting in dopaminergic hyperactivity and, ultimately, the development of psychotic symptoms [11]. Ma and Zang highlighted a positive association between this SNP and response to risperidone [55]. Additionally, rs1801028 has been linked to the efficacy of risperidone in treating negative symptoms [33]. A meta-analysis involving 27 samples and 3707 patients with SCZ concluded that Cys/Ser heterozygotes, corresponding to carriers of the C|G genotype of rs1801028, have a higher risk of developing SCZ compared to homozygotes [27]. This finding was corroborated by another study involving more than 9000 subjects [44]. A study of 120 Chinese patients showed that Cys/Ser heterozygotes respond less effectively to risperidone compared to Ser/Ser homozygotes [53]. All these findings suggest that SNPs can modulate treatment response, paving the way for more personalised approaches in managing TRS.

The serotonin receptor 5-HT2A is encoded by the HTR2A gene, a 66 kb gene located on chromosome 13q14-q21, comprising three exons [72]. Among the studied SNPs, rs6314 has been associated with the response to clozapine [31, 81] and the development of bipolar disorder (BD) [59]. Additionally, this SNP is implicated in functional changes in the prefrontal cortex and the response to olanzapine. Specifically, there is strong evidence that the T allele is linked to abnormalities in receptor expression and a minor improvement in negative symptoms during treatment [6]. Furthermore, rs6311, located within the promoter of the HTR2A gene, has been associated with SCZ [71]. A similar finding was observed in a study of a Tamil population in southern India, where the G|G genotype was found to be more prevalent among affected individuals [80]. A case–control study conducted on a German population highlighted a protective role of the A allele of rs6311 in relation to suicidal behaviour, whereas the G allele was associated with a higher risk of violent and impulsive suicidal behaviour [25].

Lastly, regarding the HTR2A SNP rs7997012, to the best of our knowledge, no studies have focused on or demonstrate a possible association between rs7997012 and TRS. However, this SNP has been linked to antidepressant treatment response in patients affected by major depressive disorder (MDD). Specifically, homozygosity for the A allele is associated with an 18% reduction in the risk of non-response to citalopram compared to G|G homozygosity [61].

As highlighted by the studies analysed thus far, each SNPs has been individually associated with either SCZ or response to pharmacological treatment.

Some studies have also explored the association of DRD2 and HTR2A haplotypes with SCZ. Gene haplotypes represent specific combinations of genetic variations that occur along the same allele. The co-occurrence of SNPs on the same allele may lead to distinct functional effects compared to those observed with individual SNPs. Thus, studying haplotypes may help to identify patterns of genetic variations associated with diseases and clinical phenotypes.

Regarding possible haplotypes in the DRD2 gene, a study involving the Northern India population found that the haplotype A2B1D2 (formed by SNPs Taq1 A, B and D) offers protection against the development of SCZ [48]. In another study conducted in Korea, the CAT haplotype (rs4648317, rs7131056 and rs4936270) was weakly associated with the development of the disease [8]. Hussain and colleagues described the presence of a CAA haplotype (rs4936270, rs7131056, e rs4648317) that was significantly associated with an increased risk of developing SCZ [38]. In contrast, for the HTR2A gene, the TC haplotype (rs6311-rs6313) has been shown to increase the risk of developing SCZ [57]. Building on these findings, our study aims to understand the potential role that specific SNPs and haplotypes may play in the development of TRS. We hypothesise that specific SNPs localised in genes encoding D2 and 5-HT2A receptors, as well as haplotypes within the HTR2A gene, might be involved in the neuropathophysiology of TRS.

In this study, we analysed the DRD2 SNP rs1801028 and the HTR2A SNPs rs6311, rs6314 and rs7997012, with the aim of identifying genomic correlates of TRS by comparing a group of TRS patients with individuals affected by other treatment-resistant mental disorders (OTRMDs) and healthy controls (HCs).

## Methods

This study followed the Principles of Human Rights adopted by the World Medical Association (WMA) at the 18th WMA General Assembly (Helsinki, Finland, June 1964). Subsequently, it amended by the 64th WMA General Assembly (Fortaleza, Brazil, October 2013). Patients were recruited between 2018 and 2023 at the Centre of Personalised Medicine, Sant’Andrea University Hospital, Rome, Italy. Patients received comprehensive information about treatment type and purpose and provided written informed consent before inclusion. All patients were assessed for diagnosis before genotyping. The local ethical committee approved the study (protocol N. 6279/2021). The Inclusion criterion was the manifestation of a treatment-resistant mental disorder. We defined TRS as the failure of two or more antipsychotic medication trials, administered in adequate doses and duration, for a patient with schizophrenia [36], and considered four weeks as an adequate trial duration [50, 70]. We defined treatment-resistant depression as the failure of two or more adequate antidepressant trials during a depressive episode in both MDD and BD [21]. OTRMDs were defined on the same basis, i.e., failure of two or more trials in OCD, anxiety disorders, and other mental disorders. Four weeks were considered an adequate trial duration, similar to TRS. All diagnoses were established according to DSM-5 criteria [1].

We established the following exclusion criteria: age < 18 years, comorbidity with substance use disorders (except nicotine dependence), neurological illnesses (major neurocognitive disorders, Parkinson’s disease, and Huntington’s chorea), and severe acute organic conditions (i.e. major cardiovascular pathologies, uncontrolled diabetes, severe toxic, infectious and metabolic disorders, malignancy, liver failure, and renal failure).

The whole sample was divided into two subgroups: the first included patients with a diagnosis of TRS, and the second patients affected by OTRMDs. We obtained the HCs sample from an online open-access database to perform comparative statistical analyses (https://www.ensembl.org/index.html, accessed on the 15th of September 2023).

### Genetic analyses

All analyses were carried out according to the manufacturer’s protocol. DNA was isolated from 200 μL of EDTA-anticoagulated peripheral blood samples using an automated nucleic acid extraction system (Qiasymphony, Qiagen, Hilden, Germany). The genomic variants rs1801028 (DRD2 gene); rs6314, rs7997012, and rs6311 (HTR2A gene)were included in a pharmacogenomic panel analysed by Next-Generation Sequencing using the IonS5 platform (ThermoFisher Scientific, Waltham, MA, USA) and the Ion AmpliSeq™ Library Kit 2.0 reaction chemistry, following the supplier’s instructions (ThermoFisher Scientific). DNA was amplified with a pool of oligonucleotide primer pairs, each designed to amplify a genomic region of interest. Then barcoded DNA libraries were prepared, and clonal amplification and pooled library sequencing were performed on the IonChef/IonS5 system using the Ion 510™ and Ion 520™ and Ion 530™ Kit–Chef (ThermoFisher Scientific).

### Statistical analyses

We used the software JASP Team (2023), JASP (Version 0.17.3) for all analyses, except for the Hardy–Weinberg equilibrium deviation test and haplotype analyses, for which we used the SNPStats online tool (https://www.snpstats.net, accessed on 24 September 2023) [75]. Additionally, we utilised the online tool “Carbocaption” (https://tools.carbocation.com/FDR, accessed on 13 July 2023) to perform the chi-square FDR correction for multiple testing.

We performed a one-sample Shapiro–Wilk test to assess the normality of the distribution of our study’s continuous variables. Then we performed descriptive statistics with the Mann–Whitney U test for the continuous variables and the chi-square (*χ*^2^) test for the categorical variables applying the False Discovery Rate (FDR) correction using the Benjamini–Hochberg procedure. We set the cut-off for statistical significance at (two-tailed) *p* < 0.05. Referring to a chi-square method, we conducted a post-hoc power analysis for the significant results with the online tool “Sample Size Calculator” version 1.061 (https://homepage.univie.ac.at/robin.ristl/samplesize.php).

## Results

The study participants consisted of 221 consecutively admitted patients (114 women and 107 men) with a mean age of 43.14 ± 17.2 years, all of whom were affected by treatment-resistant mental disorders. The sample included 39 patients with TRS and 182 patients with OTRMDs, comprising 71 with MDD, 65 with BD, 30 with OCD, 3 with anxiety disorders, and 13 with neurodevelopmental disorders.

The TRS group had a mean age of 35.05 ± 16.18 years, while the OTRMDs group had a mean age of 44.83 ± 16.96 years. The Mann–Whitney test revealed that TRS patients were significantly younger than OTRMDs patients (U = 4651.5; p = 0.001). The Shapiro–Wilk test indicated a non-normal distribution for both age and illness duration (p < 0.001 for both). Additionally, the chi-square test showed that the male gender was significantly more represented in the TRS group compared to the OTRMDs group (χ^2^ = 9.215; *p* = 0.004), as detailed in Table 1.

**Table 1:** Chi-square test, gender distribution between the TRS and OTRMDs groups

Group		Female	Male	Total
TRS	N	102	80	182
	%	56.04%	43.96%	100%
OTRMDs	N	12	27	39
	%	30.77%	69.23%	100%
Total	N	114	107	221
	%	51.58%	48.42%	100%

	Value	df	*p*
Chi-square test			
Χ^2^	8.215	1	0.004
N°	221		

The frequencies of the analysed SNPs were in Hardy–Weinberg equilibrium across the TRS (rs1801028 χ^2^ = 3.009, p = 0.082; rs6314 χ^2^ = 1.289, p = 0.256; rs7997012 χ^2^ = 1.781; p = 0.182; rs6311 χ^2^ = 0.641, p = 0.423), OTRMDs (rs1801028 χ^2^ = 0.212, p = 0.646; rs6314 χ^2^ = 0.073; p = 0.492; rs7997012 χ^2^ = 0.352; p = 0.553; rs6311 χ^2^ = 1.799, p = 0.18), and HCs (rs1801028 χ^2^ = 0.039, p = 0.844; rs6314 χ^2^ = 1.051, p = 0.305; rs7997012 χ^2^ = 0.471; p = 0.492; rs6311 χ^2^ = 0.593, p = 0.441) groups.

The chi-square test revealed that the HTR2A rs6314 A|G vs. G|G genotype was significantly more frequent in the TRS compared to the HCs group (p = 0.01; χ^2^ = 9.289; pFDR = 0.036). Additionally, the HTR2A rs7997012 G|G vs. A|A and A|G genotype was significantly more prevalent in the TRS group compared to the HCs group (p = 0.018; χ^2^ = 8.09; pFDR = 0.036). The post-hoc power analysis for the HTR2A rs6314 A|G variant revealed a power of 81.52%, and for rs7997012 G|G, a power of 82.12%, to detect the observed differences in genetic frequencies at a significance level of 0.05. The comparisons of genotype frequencies are detailed in Table 2.

**Table 2:** DRD2 and HTR2A genotype frequencies

DRD2 rs1801028	C|C	C|G	G|G	Total	χ^2^	p	p (FDR)
HCs (%)	0	4 (3.7)	103 (96.3)	107			
TRS (%)	1 (2.5)	4 (10.3)	34 (87.2)	39			
TOT (%)	1 (0.7)	8 (5.5)	137 (93.8)	146	5.211	0.074	0.099

HTR2A rs6314	A|A	A|G	G|G	Total	χ^2^	p	p (FDR)
HCs (%)	1 (0.9)	11 (10.3)	95 (88.8)	107			
TRS (%)	0	12 (30.8)	27 (69.2)	39			
TOT (%)	1 (0.7)	23 (15.7)	122 (83.6)	146	9.289	0.01*	0.036*

HTR2A rs7997012	A|A	A|G	G|G	Total	χ^2^	p	p (FDR)
HCs (%)	17 (15.9)	47 (43.9)	43 (40.2)	107			
TRS (%)	3 (7.7)	10 (25.6)	26 (66.7)	39			
TOT (%)	20 (13.7)	57 (39.0)	69 (47.3)	146	8.09	0.018*	0.036*

HTR2A rs6311	C|C	C|T	T|T	Total	χ^2^	p	p (FDR)
HCs (%)	24 (22.4)	58 (54.2)	25 (23.4)	107			
TRS (%)	11 (28.2)	17 (43.6)	11 (28.2)	39			
TOT (%)	35 (24.0)	75 (51.4)	36 (24.6)	146	1.296	0.523	0.523

Comparison between the TRS and HCs groups (Pearson χ^2^ test)

*TRS* treatment-resistant schizophrenia, *HCs* healthy controls, *FDR*: false discovery rate

*Statistically significant result

The chi-square test did not reveal significant differences in genotype frequencies between the TRS and OTRMDs groups.

HTR2A haplotypes (rs6314—rs7997012—rs6311) were not significantly associated with TRS vs. OTRMDs group membership. However, these haplotypes were globally associated with the TRS vs. HCs group membership (global haplotype association p = 0.035), although no specific haplotype emerged as a significant predictor (Table 3).

**Table 3:** HTR2A haplotype (rs6314-rs7997012-rs6311) association with TRS vs. HCs group membership

	rs6314	rs7997012	rs6311	Total	HCs	TRS	OR (95% CI)	p
1	G	G	T	0.3486	0.3449	0.3609	1.00	–
2	G	G	C	0.2336	0.2159	0.2802	1.35 (0.64–2.83)	0.43
3	G	A	C	0.2101	0.2392	0.1335	0.63 (0.28–1.42)	0.27
4	G	A	T	0.1221	0.1393	0.0716	0.59 (0.21–1.67)	0.32
5	A	G	C	0.0529	0.0403	0.0864	2.84 (0.75–10.73)	0.13
6	A	G	T	0.0327	0.0205	0.0674	3.64 (0.58–22.87)	0.17
7	A	A	C	0				
8	A	A	T	0				

Global haplotype association p-value: 0.035*

*TRS* treatment-resistant schizophrenia, *HCs* healthy controls

^*^Statistically significant result

Given the global statistical significance of the initial model, a further haplotype association analysis was conducted using haplotypes formed by all possible SNP pairs. The HTR2A (rs6314 – rs7997012) haplotypes were globally associated with TRS vs. HCs group membership (global haplotype association p = 0.0034), with the A|G haplotype (rs6314-rs7997012) significantly predicting TRS group membership (p = 0.04; OR = 2.67) (Table 4).

**Table 4:** HTR2A haplotype (rs6314-rs7997012) association with TRS vs. HCs membership

	rs6314	rs7997012	Total	HCs	TRS	OR (95% CI)	P-value
1	G	G	0.5822	0.5607	0.641	1.00	–
2	G	A	0.3322	0.3785	0.2051	0.54 (0.29–1.01)	0.054
3	A	G	0.0856	0.0607	0.1538	2.67 (1.06–6.76)	0.04*
4	A	A	0	0	0		

Global haplotype association p-value: 0.0034*

*TRS* treatment-resistant schizophrenia, *HCs* healthy controls

^*^Statistically significant result

## Discussion

In the present study, we analysed the role of SNPs in the DRD2 (rs1801028) and HTR2A (rs6314, rs7997012, rs6311) genes in TRS, focusing on the potential implications of both dopaminergic and serotoninergic neurotransmission in TRS. We compared the SNP genotypes of patients affected by TRS with two control groups: the first comprising HCs and the second consisting of patients affected by various treatment-resistant mental disorders (OTRMDs group). Subsequently, a haplotype analysis was conducted to identify potential combinations of different genomic variants associated with TRS. For the sake of clarity, the discussion will initially be divided into sections focusing on each of the relevant SNPs, analysing not only the current literature on these specific SNPs but also how our recent research contributes to understanding their role in TRS.

*DRD2 rs1801028*: This SNP results in the substitution of serine for cysteine at codon 311 [41]. Ser311 is located in the third intracellular loop of the D2 receptor, a crucial position for modulating the receptor/G-protein interaction [68]. The rs1801028 variant may lead to the formation of a new disulphide bridge, causing subsequent structural changes [2], and it also alters receptor desensitization and internalization processes [42]. Additionally, the Cys311 variant is less effective at inhibiting cyclic adenosine monophosphate (cAMP) synthesis compared to the more common Ser311 variant [13].

We observed a significant trend in the expressions of rs1801028 genotypes when comparing the TRS group to the HCs group. Our findings are partially consistent with previous studies. Two separate meta-analyses have indicated that Cys311 may represent a dominant allele [27], acting as a risk factor for SCZ [26, 28]. However, this variant also has pharmacogenomic implications. In acute psychosis, the Ser311Cys genotype is associated with a poorer response to risperidone, both in terms of psychopathological status and social functioning, compared to the Ser311Ser genotype [53]. Carriers of the Cys311 variant demonstrated an improved response to atypical APs, resulting in decreased hospitalisation and a lower incidence of treatment resistance [2, 63]. Considering the available literature, the evidence of only a trend of association in our sample may reflect the limited sample size or the specific inclusion of treatment-resistant conditions, highlighting the need for further confirmation in larger samples.

*HTR2A rs6314*: This SNP is located on exon 3 of the HTR2A gene and results in a missense mutation of the amino acid position 452 in the carboxy-terminal region, substituting histidine with tyrosine [66]. The rs6314 variant appears to alter calcium-dependent intracellular signalling pathways and the activation of phospholipases C and D [34, 65]. Carriers of the 452Tyr allele have been shown to exhibit a significant reduction in the fractional volume of temporal white matter and grey matter in the left hippocampus, left inferior temporal gyrus and bilaterally in the middle and superior temporal gyri [22]. This finding supports the previously noted involvement of rs6314 in human memory processes [15, 73]. One possible interpretation is that individuals carrying the 452Tyr allele may experience alterations in prefrontal function during cognitive tasks, a phenomenon that has been described as an endophenotype associated with genetic risk for SCZ [6]. Furthermore, this HTR2A SNP may influence neuroplasticity through the 5-HT2A receptor [58, 64], potentially involving reorganization mechanisms within glutamatergic cortical circuits as well [43].

According to our findings, the A|G genotype of rs6314 was significantly more frequent in the TRS group compared to the HCs group. However, no significant differences in its expression were observed between patients with OTRMDs and those with TRS. These results are consistent with the existing literature, which highlights the importance of rs6314 in both AP drug response and prefrontal function. Blasi et al. conducted a study on patients with SCZ and found that the G|G genotype was associated with a better response to olanzapine, while the T allele was linked to poorer prefrontal function [6]. Regarding the response to clozapine, the Tyr453 variant has been associated with treatment resistance in several studies [3–5, 56]. The substitution of histidine with tyrosine, given their different biochemical properties, may alter the tertiary structure of 5-HT2A receptor and, consequently, its function. For instance, the Tyr452 variant appears to disrupt the physiological 5-HT2A/D2 dimerization process. Interestingly, clozapine may potentially compensate for this disruption by promoting dimerization [54].

*HTR2A rs7997012*: This SNP is located in the second intron of the HTR2A gene [51]. Of the two possible nucleobases, adenine and guanine, guanine is more frequently found. Several studies have highlighted the role of rs7997012 in the response to antidepressant treatment [18, 24, 82, 86]. The A allele is associated with a higher response rate to citalopram and is more prevalent in the Caucasian population than in the African-American population, which may explain ethnic differences in response to this SSRI [61]. However, there is a notable lack of studies focusing on the response to APs and SCZ spectrum disorders.

To our knowledge, this is the first work to examine rs7997012 in the context of TRS. Our findings reveal that the case group had a significantly higher proportion of G|G genotypes compared to the HCs group, while no significant difference was observed between patients with TRS and those with OTRMDs. Most of the available literature focuses on the pharmacogenomic implications of the rs7997012 genetic marker. Evidence from a systematic review suggests that the A allele may be associated with a less tolerable profile when patients are treated with olanzapine [88]. This finding indicates that pharmacogenomic-guided dose adjustment could be beneficial for patients undergoing such treatment. In our previous study [16], the A|G heterozygous genotype was found to predict the inclusion of patients with various difficult-to-treat mental disorders in a high treatment resistance category. Whether treatment resistance arises from unique pathophysiological mechanisms or from individual variations in drug response remains unclear. However, when considered alongside our current findings, the role of rs7997012 presents promising avenues for further investigations.

*HTR2A rs6311*: This genomic variant, referred to as −1438A/G, is located in the promoter region of the HTR2A gene. The rs6311 variant has been linked to numerous neuropsychiatric phenotypes due to its role in modulating promoter activity [67]. Our investigation found no significant differences in the presence of rs6311 among the groups studied. However, both the rs6314-rs6311 and rs7997012-rs6311 haplotypes showed only a trend towards association with the TRS group compared to the HCs group. These results are only partially consistent with the existing literature. Spurlock et al. found that rs6311 is in almost complete linkage disequilibrium with the 102 T/C SNP, a silent variant in exon 1 of the HTR2A gene [77]. The 102C allele has been associated with a diagnosis of SCZ [85] and with a poor response to clozapine [4, 5]. Given that these two SNPs are in complete linkage disequilibrium, meaning that they are statistically associated with one another [30], it is plausible that rs6311 may also be implicated in these conditions. Moreover, some studies suggest that specific rs6311 configurations could be related to the SCZ risk: a meta-analysis by Lian et al. found that the −1438A allele was associated with an increased risk of SCZ in Caucasians compared to Asians [32]. Additionally, a study on the Tamil population reported a higher prevalence of the G|G genotype in SCZ patients than in HCs [80].

*Haplotype analysis*: In our discussion, we examined the results of single SNP association analyses, which identified genomic regions potentially linked to TRS. However, identifying these regions is just the beginning, as SCZ remains a complex disorder with genetic bases that are not yet fully understood [78]. This complexity is particularly pronounced in TRS. Our findings suggest that specific SNP genotypes may contribute to TRS by altering neurotransmission in the central nervous system, particularly through the 5-HT2A receptor within the serotoninergic system.

There is strong evidence supporting the role of serotonergic dysfunction in SCZ. The serotonin theory posits that overactivity of 5-HT2A receptors in glutamatergic neurons in the anterior cingulate cortex and dorsolateral frontal lobe leads to abnormal glutamate release in the ventral tegmental area. This, in turn, causes hyperactivation of mesolimbic pathways, resulting in significant dopamine release in the ventral striatum [79]. To further explore the involvement of the HTR2A gene, we conducted haplotype analyses for SNPs located within this gene. By complementing the single SNP association analysis, we aimed to identify sets of SNPs associated with TRS that can offer deeper insights into their potential neuropathological role. To the best of our knowledge, this is the first study to primarily focus on haplotype analysis and evaluate the association of rs6314, rs7997012, and rs6311 haplotypes with TRS diagnosis.

Our analysis revealed that the haplotype AG (rs6314-rs7997012) was significantly associated with TRS membership, compared with HCs. This result aligns with the single SNP association analysis, which showed that both SNPs were significantly more frequent in TRS patients.

Moreover, our findings partially align with previous studies that have investigated haplotypes including rs6311. For instance, the haplotype TC (rs6311-rs6313) has been associated with an increased risk of SCZ [57], as well as the haplotype GCC (rs6311-rs6313-rs6305) [48]. These results suggest that future studies should explore how multiple SNP effects interact with each other to uncover the underlying mechanisms of SCZ and TRS. The HTR2A gene continues to emerge as a highly significant region of interest in this context.

*Limitations and strengths*: Our study employed specific inclusion criteria for selecting patients affected by treatment-resistant mental disorders and utilised a real-world sample. However, it is important to emphasise the need further studies to validate and generalise our findings, given the varying criteria used in the literature to define treatment-resistant conditions. Another limitation of our study is the relatively small sample size. However, as the calculated power exceeded the commonly accepted threshold of 80%, we conclude that our study was well-powered to detect the observed genetic frequency differences, with a low likelihood of committing Type II errors.

## Conclusion

Certain SNPs of the HTR2A gene show a differential distribution between patients with TRS and HCs. Specifically, the HTR2A rs6314 A|G vs. G|G genotype was significantly more frequent in the TRS group compared to the HCs group. Additionally, the HTR2A rs7997012 G|G vs. A|A and A|G genotype was significantly more prevalent in the TRS vs. HCs group. Notably, our study is the first to report that the haplotype A|G (rs6314-rs7997012) predicts TRS versus HCs membership.

The serotoninergic system, along with other genetic and environmental factors, plays a significant role in the pathophysiology of SCZ. Our findings suggests that TRS does not exhibit significant differences compared to OTRMDs, indicating a potential trans-nosographic nature of treatment resistance. Furthermore, specific haplotypes may serve as predictors of TRS membership, offering valuable insights for research into the underlying pathophysiological mechanisms of SCZ and the development of genomic-guided treatment strategies in psychiatry.

## Data Availability

The data that support the findings of this study are available upon reasonable request from the following authors: Antonio Del Casale (antonio.delcasale@uniroma1.it) and Marina Borro (marina.borro@uniroma1.it).

## Abbreviations

5-HT2A: 5-Hydroxytryptamine (serotonin) 2A receptor
AP(s): Antipsychotic(s)
BD: Bipolar disorder
cAMP: Cyclic adenosine monophosphate
DRD2: Dopamine receptor D2 gene
FDA: Food and drug administration
FDR: False discovery rate
HCs: Healthy controls
HTR2A: 5-Hydroxytryptamine (serotonin) receptor 2A gene
MDD: Major depressive disorder
OCD: Obsessive–compulsive disorder
OTRMDs: Other treatment-resistant mental disorders
SCZ: Schizophrenia
SNP(s): Single nucleotide polymorphism(s)
TRRIP: Treatment response and resistance in psychosis
TRS: Treatment-resistant schizophrenia
